# Moderate Alcohol Exposure during the Rat Equivalent to the Third Trimester of Human Pregnancy Alters Regulation of GABA_A_ Receptor-Mediated Synaptic Transmission by Dopamine in the Basolateral Amygdala

**DOI:** 10.3389/fped.2014.00046

**Published:** 2014-05-27

**Authors:** Marvin Rafael Diaz, Karick Jotty, Jason L. Locke, Sara R. Jones, Carlos Fernando Valenzuela

**Affiliations:** ^1^Department of Neurosciences, University of New Mexico Health Sciences Center, Albuquerque, NM, USA; ^2^Department of Physiology and Pharmacology, Wake Forest School of Medicine, Winston-Salem, NC, USA

**Keywords:** fetal, alcohol, BLA, dopamine, GABA, electrophysiology, prenatal, homeostasis

## Abstract

Fetal ethanol (EtOH) exposure leads to a range of neurobehavioral alterations, including deficits in emotional processing. The basolateral amygdala (BLA) plays a critical role in modulating emotional processing, in part, via dopamine (DA) regulation of GABA transmission. This BLA modulatory system is acquired during the first 2 weeks of postnatal life in rodents (equivalent to the third trimester of human pregnancy) and we hypothesized that it could be altered by EtOH exposure during this period. We found that exposure of rats to moderate levels of EtOH vapor during the third trimester-equivalent [postnatal days (P) 2–12] alters DA modulation of GABAergic transmission in BLA pyramidal neurons during periadolescence. Specifically, D1R-mediated potentiation of spontaneous inhibitory postsynaptic currents (IPSCs) was significantly attenuated in EtOH-exposed animals. However, this was associated with a compensatory decrease in D3R-mediated suppression of miniature IPSCs. Western blot analysis revealed that these effects were not a result of altered D1R or D3R levels. BLA samples from EtOH-exposed animals also had significantly lower levels of the DA precursor (L-3,4-dihydroxyphenylalanine) but DA levels were not affected. This is likely a consequence of reduced catabolism of DA, as indicated by reduced levels of 3,4-dihydroxyphenylacetic acid and homovanillic acid in the BLA samples. Anxiety-like behavior was not altered in EtOH-exposed animals. This is the first study to demonstrate that the modulatory actions of DA in the BLA are altered by developmental EtOH exposure. Although compensatory adaptations were engaged in our moderate EtOH exposure paradigm, it is possible that these are not able to restore homeostasis and correct anxiety-like behaviors under conditions of heavier EtOH exposure. Therefore, future studies should investigate the potential role of alterations in the modulatory actions of DA in the pathophysiology of fetal alcohol spectrum disorders.

## Introduction

Fetal exposure to ethanol (EtOH) is a leading cause of mental retardation in the world and can lead to a myriad of complications known as Fetal Alcohol Spectrum Disorders (FASDs). FASDs are a major public health problem with an estimated prevalence of 1–5% in the United States (1). FASDs can range from severe mental retardation and facial dysmorphologies to more subtle cognitive/behavioral deficits in the absence of morphological alterations. Importantly, ~20% of children and adolescents with FASDs suffer from emotional processing deficits, such as anxiety, that can manifest into adverse long-term outcomes and poor social adjustment (2). The interventions available to treat emotional processing deficits in patients with FASDs are only partially effective and this is a consequence of our limited understanding of the cellular and molecular underpinnings of these disorders (2).

The amygdala is a key mediator of emotional processing in humans and rodents. The basolateral amygdala (BLA) functions to locally process sensory and cortical information required to generate appropriate emotional responses (3). BLA glutamatergic pyramidal neuron activity is positively correlated with anxiety-like behaviors (4) and excitability of these neurons is regulated by GABAergic interneurons. Stress-inducing novel experiences increase dopamine (DA) release in the BLA (5, 6). In humans, this DA surge has been suggested to regulate amygdala function depending on environmental stimuli (7). In rodents, DA fibers innervate both pyramidal neurons (8) and interneurons (9, 10). Importantly, DA can bi-directionally regulate GABA_A_ receptor (GABA_A_R)-dependent synaptic transmission by (1) increasing local interneuron excitability via type-1 DA receptors (D1Rs) (11), and (2) decreasing quantal GABA release onto pyramidal neurons via type-3 receptors (D3Rs) (12). Consistent with the actions of D1Rs in the BLA, it has been demonstrated that microinjection of a D1R antagonist into the BLA results in anxiogenesis (13). In contrast, microinjections of D3R antagonists directly into the BLA or D3R deficiency reduces anxiety-like behaviors (12, 14).

A common drinking pattern in pregnant women is to abstain from EtOH during the first two trimesters, followed by consumption during the third trimester (15). Low-to-moderate EtOH exposure during late pregnancy has been associated with increased incidence of anxiety disorders in offspring during childhood (16). During the human third trimester of pregnancy, neuronal circuits undergo significant refinement, and a number of neurotransmitter systems are acquired. Similar neuronal processes occur in rodents in the first two postnatal weeks, particularly the development of GABAergic interneurons (17) and DA innervation (18) within the BLA. Exposure to EtOH during this period of rodent development has been used to model human exposure during the third trimester of pregnancy. The objective of this study was to examine whether exposure of rats to moderate EtOH levels during the third trimester-equivalent period impairs D1R- and D3R-dependent modulation of GABA_A_R-mediated synaptic transmission at pyramidal neurons in the BLA. We also characterized the effect of EtOH on levels of DA, its precursor, and its metabolites in this brain region. Finally, we assessed anxiety-like behaviors in peri-adolescent animals.

## Materials and Methods

Unless indicated, all drugs and chemicals were from Sigma-Aldrich (St. Louis, MO, USA).

### Animals

All animal procedures were approved by the UNM-Health Sciences Center Institutional Animal Care and Use Committee and conformed to NIH Guidelines. Pregnant Sprague-Dawley rats were obtained from Harlan (Indianapolis, IN, USA) and arrived at gestational day 12–16. Dams were individually housed, received food and water *ad libitum*, and had a plastic hut in the cage to reduce stress.

### Ethanol vapor chamber exposure

To model third trimester-equivalent EtOH exposure, we exposed dams with their pups from postnatal day (P) 2 to P12 from 10 a.m. to 2 p.m. daily using vapor inhalation chambers (19, 20). EtOH vapor/air mixture was equilibrated to reach ~3–3.5 g/dL measured with a breathalyzer (Intoximeters, St. Louis, MO, USA). During the 10 days of exposure, animals were handled only once on P5 for bedding and cage change, at which time litters were culled to 8–12 pups. After exposure, offspring were allowed to mature to P40–P50 for electrophysiology experiments, tissue collection, or behavioral testing. Animals were also weighed at P40–P50. Only males were used for this study.

Pup serum EtOH concentrations (SECs) were determined by taking pups immediately after the exposure on P6 and P12. Animals were anesthetized with ketamine (250 mg/kg i.p.), decapitated, and trunk blood was collected and mixed with 6.6% perchloric acid (50 μl of blood and 450 μl of perchloric acid). Samples were centrifuged at a relative centrifugal force of 3000 for 15 min at 4°C. SECs were measured in the supernatants using a standard alcohol dehydrogenase-based assay, as previously described (21).

### BLA slice electrophysiology

For slice preparation, animals were sacrificed by rapid decapitation under deep anesthesia with ketamine (250 mg/kg i.p.) and brains were quickly removed and submerged for 2 min in cold sucrose artificial cerebro spinal fluid (aCSF) containing (in millimolar): 220 sucrose, 2 KCl, 1.25 NaH_2_PO_4_, 26 NaHCO_3_, 12 MgSO_4_, 10 glucose, 0.2 CaCl_2_, and 0.43 ketamine, pre-equilibrated with 95% O_2_/5% CO_2_. Coronal brain slices containing the BLA (250 μm) were prepared using a vibrating tissue slicer (Leica Microsystems, Bannockburn, IL, USA). Immediately following this procedure, slices were placed in a chamber containing normal aCSF and allowed to recover for 40 min at 35–36°C followed by storage at 22°C. Normal aCSF contained (in millimolar): 126 NaCl, 2 KCl, 1.25 NaH_2_PO_4_, 26 NaHCO_3_, 10 glucose, 1 MgSO_4_, 2 CaCl_2_, and 0.4 ascorbic acid and was continuously equilibrated with 95% O_2_/5% CO_2_.

For whole-cell patch-clamp electrophysiological recordings, neurons were visualized using infrared-differential interference contrast microscopy and recordings were performed with an Axopatch 200B amplifier (Molecular Devices, Sunnyvale, CA, USA). BLA pyramidal neurons were identified on the basis of their morphology (large/pyramidal-shaped) and capacitance ≥150 pF. To record spontaneous inhibitory postsynaptic currents (sIPSCs), a KCl-based internal solution was used (12). In some experiments, tetrodotoxin (TTX; 1 μM; Tocris, Ellisville, MO, USA) was added to the aCSF to block action potential-dependent events and record miniature IPSCs (mIPSCs). IPSCs were isolated by blocking AMPA and NMDA receptors using kynurenic acid (1 mM) and dl-APV (50 μM; Tocris). The holding potential was −70 mV. During application of glutamate antagonists, neurons were allowed to equilibrate for at least 5 min prior to beginning an experiment. Data were acquired in gap-free mode at 10 kHz and filtered at 2 kHz. Only recordings where the access resistance changed <20% were kept for analysis.

### Western immunoblotting

BLA tissue was micro-dissected from coronal slices from Air and EtOH animals. These were prepared as described above, collected immediately after slicing, and flash frozen in liquid nitrogen. Tissue was then sonicated in homogenization buffer (0.1 g tissue/1 mL of buffer) containing: 25 mM HEPES (pH 7.4), 500 mM NaCl, 2 mM EDTA, 1 mM dithiothreitol, 0.1% Tween-20, 1 mM phenylmethanesulfonyl fluoride, 20 mM NaF, 1% v/v phosphatase cocktail (Sigma-Aldrich, Cat # P2850), 5 μM cyclosporine A, and 1 Complete Mini Protease tablet/10 mL (Cat # 11836153001, Roche Diagnostics, Indianapolis, IN, USA), and stored in 10 μL aliquots at −80°C. The protein concentration was determined by the Bradford Method (BioRad, Hercules, CA, USA) using bovine serum albumin as a standard.

Samples were mixed with sodium dodecyl sulfate polyacrylamide gel electrophoresis sample buffer (Final concentration: 250 mM Tris–HCl (pH 6.8), 10% sodium dodecyl sulfate, 30% glycerol, 5% β-mercaptoethanol, and 0.02% bromophenol blue), and boiled at 95°C for 5 min. Samples were loaded at a concentration of 10 μg per lane. Control experiments demonstrated that this concentration of protein was within the linear dynamic range for the western blot assay (not shown). Electrophoresis was performed in 4–15% Tris–HCl precast gels (BioRad) at 140 V for 60 min at 4°C. Proteins were blotted onto polyvinylidene fluoride membranes (0.4 μm pore size) at 100 V for 60 min at 4°C. Non-specific binding was blocked with Odyssey Blocking Buffer (Li-Cor, Lincoln, NE, USA) for 1 h at room temperature and probed overnight at 4°C with either of the following specific primary antibodies: anti-D1R antibody (1:1000, Cat # 20066; Abcam, Cambridge, England) or anti-D3R antibody (1:500, Cat # AB1786P, Millipore, Temecula, CA, USA), and mouse anti-β-actin monoclonal antibody (1:50000, Sigma-Aldrich). Visualization and relative protein densities were quantified using an Infrared Imaging System (Odyssey, LI-COR System). Membranes were then incubated for 20 s in 0.025% (w/v) Coomassie Blue R-250, 40% methanol, and 7% acetic acid in water and washed overnight with a de-staining solution containing 50% methanol and 10% acetic acid. Membranes were scanned and Coomassie staining quantified using ImageJ 1.46r software (22). For each sample, the protein expression was normalized to β-actin or an average of the intensity of randomly selected Coomassie-stained bands.

### Tissue content of DA and related analytes

BLA-containing slices were prepared as the western immunoblotting experiments and the BLA was immediately micro-dissected and snap-frozen in liquid nitrogen. Samples were homogenized in 250 μL of 0.1 M HClO_4_ and the protein concentration was determined by the bicinchoninic acid method (Thermo Scientific, Rockford, IL, USA). Extracts were centrifuged and supernatants were removed and analyzed for DA, its precursor l-3,4-dihydroxyphenylalanine (l-DOPA), and its metabolites 3,4-dihydroxyphenylacetic acid (DOPAC) and homovanillic acid (HVA) using high performance liquid chromatography (HPLC) coupled to electrochemical detection at +220 mV (ESA Inc., Chelmsford, MA, USA). Analytes were separated on a Luna 100 mm × 3.0 mm C_18_ 3 μm reverse phase HPLC column (Phenomenex, Torrance, CA, USA). The mobile phase for l-DOPA consisted of 10 μM NaCl, 46 mM NaH_2_PO_4_, 172 μM sodium octyl sulfate, 100 μM EDTA, 10% methanol, pH to 2.6 before adding the methanol and sodium octyl sulfate. For the other analytes, the mobile phase consisted of 50 mM citric acid, 90 mM NaH_2_PO_4_, 1.7–2.0 mM 1-octanesulfonic acid, 50 μM EDTA, 10–12% C_2_H_3_N, and 0.3% triethylamine (pH 3.0). Analytes were quantified using PowerChrom software (eDAQ Inc, Colorado Springs, CO, USA) and a standard calibration curve.

### Elevated plus maze

Anxiety-like behavior was assessed using an elevated plus-maze apparatus similar to that originally described (23) with incandescent lighting (~13 lux at junction). The maze consisted of two open arms and two closed arms (50.8 cm long in all cases). The open arms had a ledge 1 cm high to prevent the animals from slipping off the edge. The closed arms were surrounded by walls 30.5 cm tall. The plus-maze platform was elevated 50.8 cm above the floor. Animals were allowed to acclimate to ambient lighting and noise in the testing room for 1 h. During testing, animals were allowed to freely move in the maze for 5 min. After testing, animals were not returned to the home cage until all animals from a cage were tested to prevent exposure to stress from the tested animal (24). Between animals, the apparatus was cleaned with 70% EtOH and thoroughly dried. Animal activity was video recorded, and time spent in the open arms, closed arms, and junction, and the number of times an animal engaged in a stretch attend posture were recorded by an investigator blinded to the experimental condition of the subject.

### Data analyses

Electrophysiology recordings were initially analyzed with MiniAnalysis (Synaptosoft, Decatur, GA, USA). All data were statistically analyzed with Prizm 5 (Graphpad, San Diego, CA, USA). Initially, data were analyzed with the Pearson omnibus normality test. Only data that followed a normal distribution were analyzed using parametric tests. A *p* < 0.05 was considered to be statistically significant. Unless indicated, the experimental unit used for all statistical analyses was an animal (i.e., results obtained with all the slices from a single animal were averaged to yield a unit of determination).

## Results

### Characterization of third trimester-equivalent ethanol vapor model

To model EtOH exposure during the third trimester-equivalent, dams and pups were exposed to vaporized EtOH for 4 h per day from P2 to P12. This exposure resulted in pup SECs of 22.7 ± 1.4 mM (*n* = 16; as a reference, the legal intoxication limit in the U.S. is 17.4 mM = 0.08 g/dL); this paradigm attempted to model human exposure to relatively moderate levels of EtOH (25). There were no significant differences in pup weight between the Air and EtOH groups at the time of the electrophysiology experiments (average pup weight at P40-P50: Air = 166.6 ± 5.74 g, *n* = 8; EtOH = 158.2 ± 7.12 g, *n* = 11; Mann–Whitney *U* = 36.50, sum of ranks = 87.50, 102.5; *p* > 0.05 by Mann–Whitney test).

### Ethanol exposure did not affect basal GABAergic transmission in pyramidal neurons

**Figure 1:**
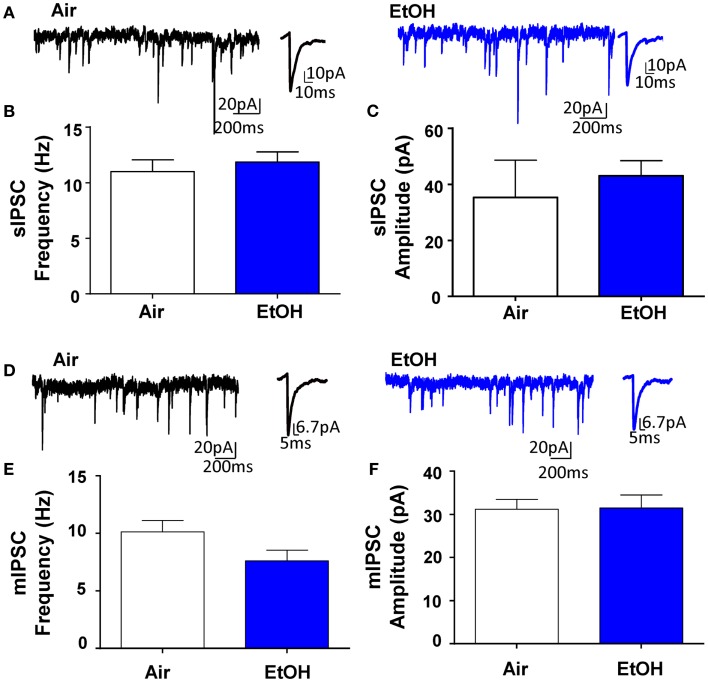
**Ethanol exposure does not affect basal spontaneous inhibitory postsynaptic currents (sIPSCs) or miniature IPSCs (mIPSCs) in the BLA**. **(A)** Exemplar compressed traces of sIPSC recordings obtained under baseline conditions from Air (black) and EtOH (blue) exposed animals. Exemplar expanded scale traces of averaged sIPSCs are shown adjacent to compressed traces. Basal sIPSC **(B)** frequency and **(C)** amplitude were not significantly different between Air- and EtOH-exposed animals (*n* = 8 animals from 5 Air and 5 EtOH litters, *p* > 0.05 by unpaired *t*-test). **(D)** Exemplar compressed traces of mIPSC recordings obtained under baseline conditions (in the presence of 1 μM TTX) from Air (black) and EtOH (blue) exposed animals. Exemplar expanded scale traces of averaged mIPSCs are shown adjacent to compressed traces. Basal mIPSC **(E)** frequency and **(F)** amplitude were not significantly different between Air- and EtOH-exposed animals (*n* = 8 animals from 5 Air and 6 EtOH litters, *p* > 0.05 by unpaired *t*-test).

Whole-cell patch-clamp electrophysiological recordings revealed that EtOH exposure had no effect on pyramidal neuron membrane capacitance (Air = 231.50 ± 19.49 pF, *n* = 10; EtOH = 222.10 ± 8.97 pF, *n* = 10; *t* = 0.44, df = 18, *p* > 0.05 by unpaired *t*-test) or membrane resistance (Air = 140.90 ± 30.05 MΩ, *n* = 10; EtOH = 123.40 ± 10.77 MΩ, *n* = 10; Mann–Whitney *U* = 46, sum of ranks = 109, 101, *p* > 0.05 by Mann–Whitney test). There was also no effect of EtOH exposure on basal sIPSC frequency (Figures 1A,B; *n* = 8, *t* = 0.63, df = 14, *p* > 0.05 by unpaired *t*-test) or amplitude (Figures 1A,C; *n* = 8, Mann–Whitney *U* = 30, sum of ranks = 66, 70, *p* > 0.05 by Mann–Whitney test). Similarly, EtOH exposure did not alter basal mIPSC frequency (Figures 1D,E; *n* = 8, *t* = 1.84, df = 14, *p* > 0.05 by unpaired *t*-test) or amplitude (Figures 1D,F; *n* = 8, *t* = 0.07, df = 14, *p* > 0.05 by unpaired *t*-test). In a different subset of pyramidal neurons from Air-exposed animals, we measured the effect of TTX on GABAergic transmission and found that TTX did not significantly alter sIPSC frequency (TTX-induced inhibition: 14.93 ± 9.56%, *n* = 7, *t* = 1.56, df = 6, *p* > 0.05 by one sample *t*-test compared to 0), but did significantly decrease sIPSC amplitude (TTX-induced inhibition: 16.30 ± 2.88%, *n* = 7, *t* = 5.66, df = 6, *p* < 0.01 by one sample *t*-test compared to 0). We confirmed that all events were blocked by the GABA_A_R antagonist, gabazine (*n* = 5; 50 μM, Tocris).

### Ethanol exposure attenuated D1R-mediated potentiation of sIPSCs in pyramidal neurons

**Figure 2:**
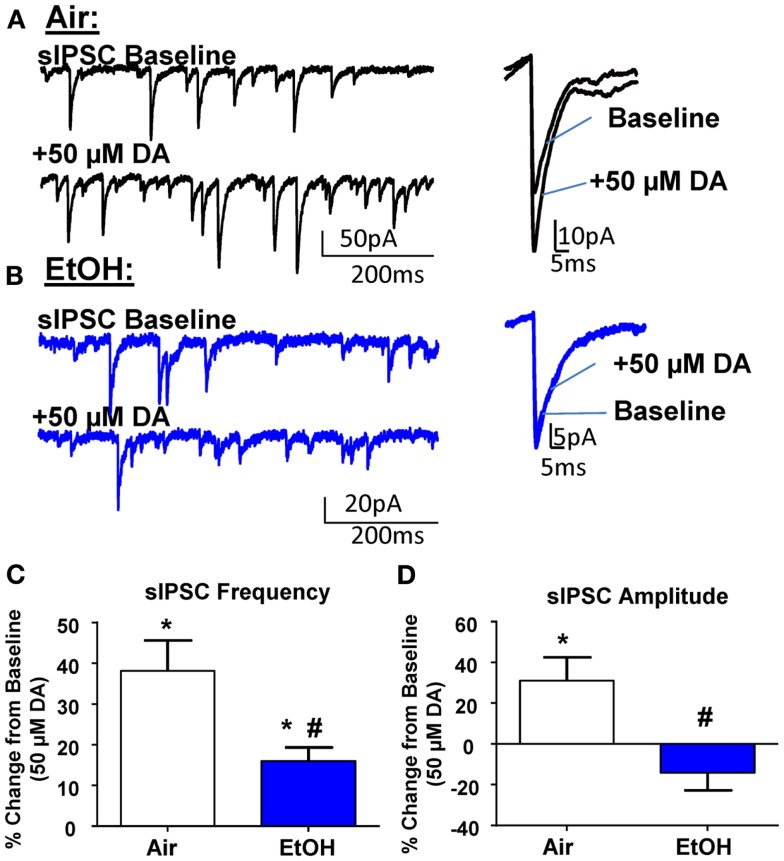
**Ethanol exposure impairs D1R-mediated facilitation of GABA_A_ receptor-mediated spontaneous inhibitory postsynaptic currents (sIPSCs) in the BLA**. Exemplar compressed traces of sIPSC recordings obtained under baseline conditions and in the presence of 50 μM dopamine (DA) in slices from **(A)** Air- and **(B)** EtOH-exposed animals. Exemplar expanded scale traces of averaged sIPSCs are shown adjacent to compressed traces. **(C)** 50 μM DA significantly increased sIPSC frequency in Air animals (*p < 0.01 by one sample *t*-test compared to 0) and EtOH animals (*p < 0.01 by one sample *t*-test compared to 0). The DA-induced increase in sIPSC frequency was significantly attenuated in EtOH compared to Air-exposed animals (^#^*p* < 0.05 by unpaired *t*-test). **(D)** sIPSC amplitude was significantly increased by 50 μM DA in Air (*p < 0.05 by one sample *t*-test compared to 0), but not EtOH-exposed animals (*p* > 0.05 by one sample *t*-test compared to 0). The DA-induced potentiation of sIPSC amplitude was significantly blunted in the EtOH compared to Air-exposed animals (^#^*p* < 0.05 by Mann–Whitney test). *N* = 8 animals from 8 Air and 7 EtOH litters.

In agreement with the literature (11, 26), application of DA (50 μM) significantly increased sIPSC frequency in Air-treated animals (Figures 2A,C; *n* = 8, *t* = 5.15, df = 7, *p* < 0.01 by one sample *t*-test compared to 0). In EtOH-treated animals DA also significantly increased sIPSC frequency (Figures 2B,C; *n* = 8, *t* = 4.78, df = 7, *p* < 0.01 by one sample *t*-test compared to 0); however, this effect was significantly blunted compared to Air-exposed animals (Figure 2C; *t* = 2.73, df = 14, *n* = 8, *p* < 0.05 by unpaired *t*-test). Application of DA (50 μM) significantly increased sIPSC amplitude in Air-treated animals (Figures 2A,D; *n* = 8, *t* = 2.71, df = 7, *p* < 0.05 by one sample *t*-test compared to 0). In contrast, the sIPSC amplitude was not altered by DA in the EtOH-exposed slices (Figures 2B,D; *n* = 8, *t* = 1.61, df = 7, *p* > 0.05 by one sample *t*-test compared to 0). The DA-induced effect on sIPSC amplitude was significantly different between Air- and EtOH-treated animals (Figure 2D; Mann–Whitney *U* = 8, sum of ranks = 92, 44, *n* = 8, *p* < 0.05 by Mann–Whitney test).

The DA-induced increase of sIPSC frequency has been shown to be mediated by activation of D1Rs, which increase the excitability of local interneurons (11, 26, 27). To confirm these findings, we first examined the effect of the D1R antagonist, SCH23390 (10 μM), on GABAergic transmission from Air-exposed animals and found that this agent alone did not induce a significant change in basal sIPSC frequency (change from baseline: 12.28 ± 8.70%; *n* = 5, *t* = 1.41, df = 4, *p* > 0.05 compared to 0 by one sample *t*-test) or amplitude (change from baseline: 13.96 ± 13.06%; *n* = 5, *t* = 1.07, df = 4, *p* > 0.05 compared to 0 by one sample *t*-test). SCH23390 also did not alter mIPSC frequency (change from baseline: −5.37 ± 6.24%; *n* = 5, *t* = 0.86, df = 4, *p* > 0.05 compared to 0 by one sample *t*-test) or amplitude (change from baseline: −12.12 ± 11.13%; *n* = 5, *t* = 1.08, df = 4, *p* > 0.05 compared to 0 by one sample *t*-test). In the presence of SCH23390, DA did not significantly change sIPSC frequency in either Air-exposed (change from baseline: −14.46 ± 8.57%; *n* = 8, *t* = 1.69, df = 7, *p* > 0.05 compared to 0 by one sample *t-*test) or EtOH-exposed (change from baseline: 2.12 ± 2.84%; *n* = 8, *t* = 0.74, df = 7, *p* > 0.05 compared to 0 by one sample *t-*test). Similarly, in the presence of SCH23390, DA did not significantly change sIPSC amplitude in Air (change from baseline: −0.87 ± 10.48%; *n* = 8, *t* = 0.08, df = 7, *p* > 0.05 compared to 0 by one sample *t-*test) or EtOH-treated animals (change from baseline: −13.29 ± 9.78%; *n* = 8, *t* = 1.36, df = 7, *p* > 0.05 compared to 0 by one sample *t-*test).

It is important to note that micromolar concentrations of DA have been shown to activate noradrenergic receptors, specifically α1 (28). Furthermore, norepinephrine-mediated activation of α1-adrenoreceptors in the BLA can increase GABA transmission (29), similar to the D1R-mediated effect on sIPSCs. Therefore, we investigated the effect of DA (50 μM) on sIPSCs in the presence of the selective α1 antagonist, doxazosin (25 μM; Tocris), and found that DA still significantly increased sIPSC frequency (change from baseline: 27.58 ± 5.19%; *n* = 6, *t* = 5.31, df = 5, *p* < 0.05 compared to 0 by one sample *t*-test) without altering sIPSC amplitude (change from baseline: −12.90 ± 11.07%; *n* = 6, *t* = 1.16, df = 5, *p* < 0.05 compared to 0 by one sample *t*-test). Importantly, there was no significant difference between the effect of DA and DA + doxazosin on sIPSC frequency (*t* = 1.09, df = 12, *p* > 0.05 by unpaired *t*-test). However, there was a significant difference between the effect of DA (*n* = 8) and DA + doxazosin on sIPSC amplitude (*n* = 6, Mann–Whitney *U* = 7, sum of ranks = 77, 28, *p* < 0.05 by Mann–Whitney test).

### Exposure to ethanol impaired D3R-mediated suppression of mIPSCs in pyramidal neurons

**Figure 3:**
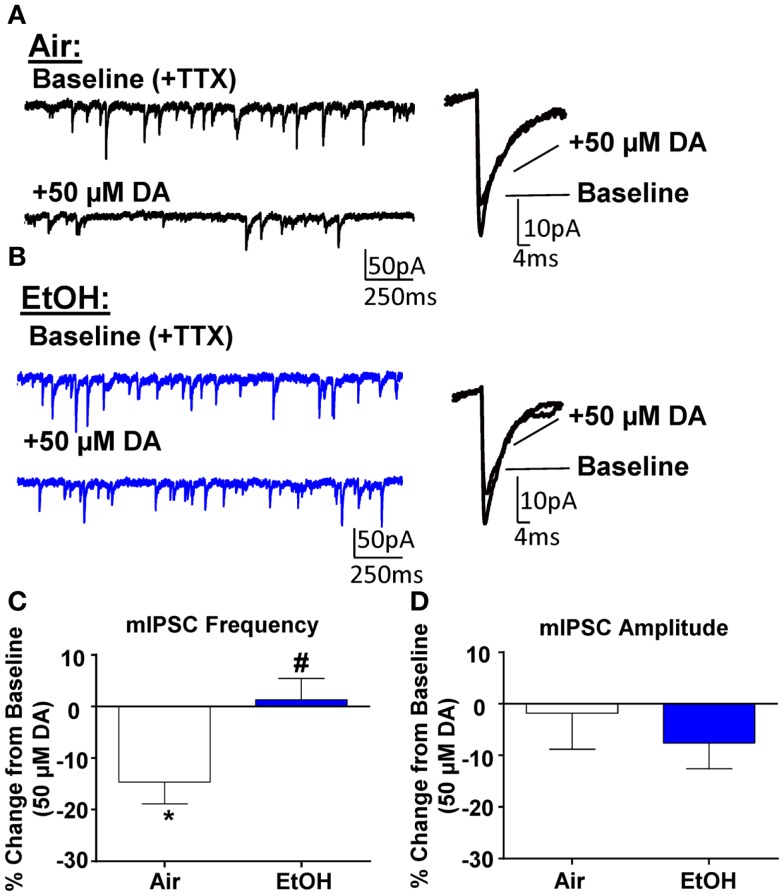
**Ethanol exposure impairs D3R-mediated suppression of GABA_A_ receptor-mediated miniature inhibitory postsynaptic currents (mIPSCs) in the BLA**. Exemplar compressed traces of mIPSC recordings obtained under Baseline conditions (in the presence of 1 μM TTX) and in the presence of 50 μM dopamine (DA) in slices from **(A)** Air- and **(B)** EtOH-exposed animals. Exemplar expanded scale traces of averaged mIPSCs are shown adjacent to these. **(C)** 50 μM DA significantly decreased mIPSC frequency in Air animals (*p < 0.05 by one sample *t*-test compared to 0), but not in EtOH animals (*p* > 0.05 by one sample *t*-test compared to 0). The DA-induced decrease in mIPSC frequency was significantly attenuated in EtOH compared to Air-exposed animals (^#^*p* < 0.05 by unpaired *t*-test). **(D)** mIPSC amplitude was not altered by 50 μM DA in either Air or EtOH-exposed animals. *N* = 8 animals from 6 Air and 5 EtOH litters.

Application of DA (50 μM) significantly decreased mIPSC frequency in Air-treated animals (Figures 3A,C; *n* = 8, *t* = 3.44, df = 7, *p* < 0.05 compared to 0 by one sample *t*-test). A similar effect had previously been shown using a selective D3R agonist, which can reduce quantal GABA release from local interneurons (12). Interestingly, in EtOH-treated animals, DA did not significantly alter mIPSC frequency (Figures 3B,C; *n* = 8, *t* = 0.31, df = 7, *p* > 0.05 compared to 0 by one sample *t*-test); and this effect was significantly different compared to Air-exposed animals (Figure 3C; *t* = 2.67, df = 14, *n* = 8, *p* < 0.05 by unpaired *t*-test). Application of DA (50 μM) did not significantly alter mIPSC amplitude in either Air- (Figure 3D; *t* = 0.27, df = 7, *p* > 0.05 compared to 0 by one sample *t*-test) or EtOH-treated animals (Figure 3D; *t* = 1.52, df = 7, *n* = 8, *p* > 0.05 compared to 0 by one sample *t*-test) and this effect of DA on mIPSC amplitude was not significantly different between Air- and EtOH-treated slices (Figure 3D; *t* = 0.67, df = 14, *n* = 8, *p* > 0.05 by unpaired *t*-test).

To determine if there was a tonic D3R activation, we first examined the effect of the selective D3R antagonist, GR103691 (1 μM; Tocris), on GABA transmission. After statistically identifying and removing one outlier (Grubbs outlier test), we found that GR103691 alone did not alter sIPSC frequency (change from baseline: 16.36 ± 14.61; *n* = 5, *t* = 1.12, df = 4, *p* > 0.05 compared to 0 by one sample *t*-test) or amplitude (change from baseline: −13.40 ± 10.98%; *n* = 6, *t* = 1.22, df = 4, *p* > 0.05 compared to 0 by one sample *t*-test). Likewise, GR103691 alone did not affect mIPSC frequency (change from baseline: −3.35 ± 4.04; *n* = 5, *t* = 0.82, df = 4, *p* > 0.05 compared to 0 by one sample *t*-test) or amplitude (change from baseline: 6.27 ± 7.44%; *n* = 5, *t* = 0.84, df = 4, *p* > 0.05 compared to 0 by one sample *t*-test). Consistent with previous reports (12), in the presence of GR103691 (1 μM), DA did not significantly change mIPSC frequency (DA-induced change: 10.38 ± 6.05%, *n* = 8, *t* = 1.72, df = 7, *p* > 0.05 compared to 0 by one sample *t-*test) or amplitude (DA-induced change: −0.76 ± 4.19%, *n* = 8, *t* = 0.18, df = 7, *p* > 0.05 compared to 0 by one sample *t-*test) in Air-exposed animals.

### D1R and D3R expression was not affected by ethanol exposure

**Figure 4:**
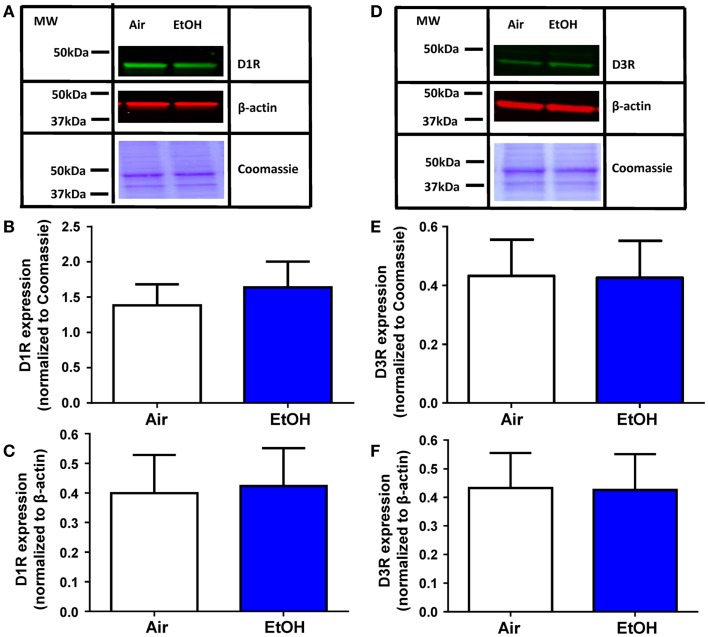
**D1R and D3R expression in the BLA is not affected by ethanol exposure**. **(A)** Exemplar western blots illustrating levels of D1R and β-actin levels in samples from Air- and EtOH-exposed rats. Also shown are Coommassie stained bands. Graphic quantification of D1R expression normalized to **(B)** Coomassie-stained bands and **(C)** β-actin. D1R expression was not altered by EtOH exposure in either analysis. **(D)** Same as in **(A)** but for D3R. Graphic quantification of D3R expression normalized to **(E)** Coomassie-stained bands and **(F)** β-actin. D3R expression was not altered by EtOH exposure in either analysis. *N* = 8 animals from 4 Air and 4 EtOH litters for all western blots.

The EtOH-mediated effects on D1R and D3R function could be explained by a decrease in the levels of these receptors. Western blot analysis of total D1R expression within the BLA (Figure 4A) showed no effect of EtOH exposure when normalized to either Coomassie-stained bands (Figure 4B; Mann–Whitney *U* = 28, sum of ranks = 64, 72, *n* = 8, *p* > 0.05 by Mann–Whitney test) or β-actin (Figure 4C; *t* = 0.13, df = 14, *n* = 8, *p* > 0.05 by unpaired *t*-test). Similarly, D3R expression within the BLA (Figure 4D) was not significantly altered by EtOH exposure when normalized to Coomassie-stained bands (Figure 4E; *t* = 0.04, df = 14, *n* = 8, *p* > 0.05 by unpaired *t*-test) or β-actin (Figure 4F; *t* = 0.18, df = 14, *n* = 8, *p* > 0.05 by unpaired *t*-test).

### Exposure to ethanol decreased DA metabolite levels in the BLA

**Figure 5:**
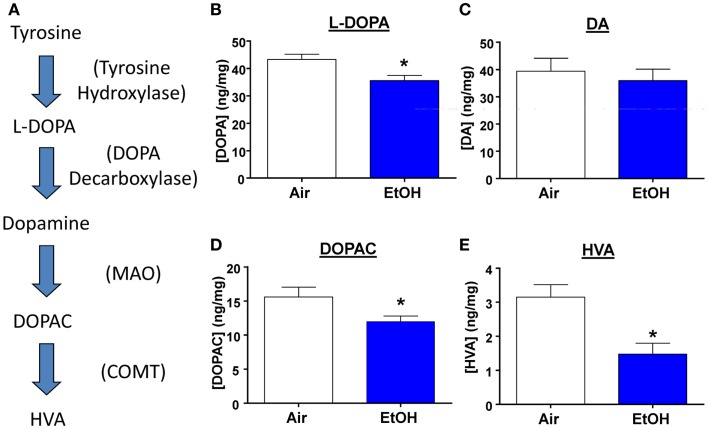
**l-DOPA and DA metabolite levels are decreased by ethanol exposure**. **(A)** Schematic representation of the DA synthesis pathway. Ethanol exposure significantly reduced levels of **(B)** l-DOPA (*p < 0.05 by unpaired *t*-test), but did not alter **(C)** DA levels (*p* > 0.05 by unpaired *t*-test). EtOH exposure also significantly reduced levels of **(D)** DOPAC (*p < 0.05 by Mann–Whitney test), and **(E)** HVA (*p < 0.05 by Mann–Whitney test). *N* = 8 animals from 4 Air and 4 EtOH litters.

Several studies have shown that developmental exposure to EtOH leads to decreased DA levels throughout the brain (30). Therefore, we measured levels of DA, its precursor, and its metabolites from micro-dissected BLA samples (Figure 5A). We found that EtOH-exposed animals had significantly reduced levels of the DA precursor, l-DOPA (Figure 5B; *n* = 8, *t* = 2.92, df = 14, *p* < 0.05 by unpaired *t*-test). However, DA levels were unaltered by EtOH exposure (Figure 5C; *n* = 8, *t* = 0.54, df = 14, *p* > 0.05 by unpaired *t*-test), This is probably a consequence of reduced degradation of DA; levels of its metabolites were significantly reduced in the samples from the EtOH group (DOPAC Figure 5D; *n* = 8, Mann–Whitney *U* = 11, sum of ranks = 89, 47, *p* < 0.05 by Mann–Whitney test; HVA; Figure 5E; *n* = 8, Mann–Whitney *U* = 10, sum of ranks = 90, 46, *p* < 0.05 by Mann–Whitney test).

### Anxiety-like behavior was not affected by ethanol exposure in periadolescence

**Figure 6:**
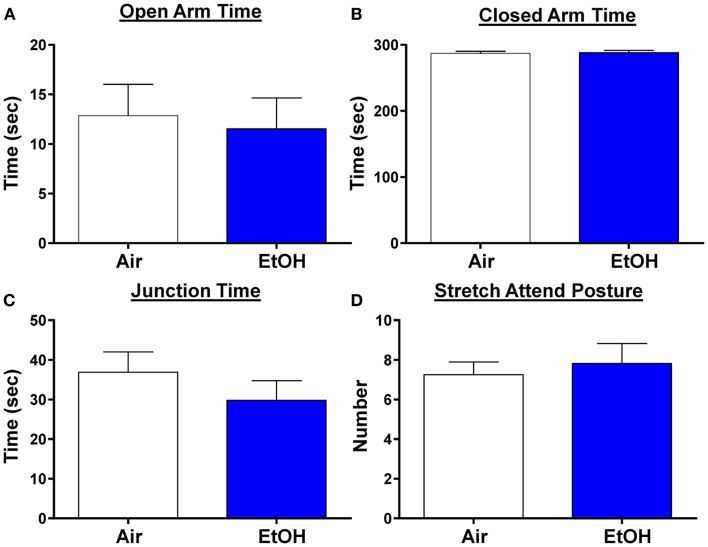
**Ethanol exposure did not alter anxiety-like behavior on the elevated plus maze**. Exposure to ethanol had no effect on **(A)** open arm time (*p* > 0.05 by unpaired *t*-test), **(B)** closed arm time (*p* > 0.05 by unpaired *t*-test), **(C)** junction time (*p* > 0.05 by unpaired *t*-test), or **(D)** number of times animals engaged in stretch attend posture (*p* > 0.05 by unpaired *t*-test). *N* = 8 animals from 3 Air litters and 11 animals from 5 EtOH litters.

We next examined anxiety-like behavior using the elevated plus maze. We did not find any changes in the time spent in the open arms, a reliable measure of anxiety-like behavior (Figure 6A; *n* = 8 Air and 11 EtOH, *t* = 0.29, df = 17, *p* > 0.05 by unpaired *t*-test). Likewise, there was no difference in the time spent in the closed arms (Figure 6B; *n* = 8 Air and 11 EtOH, *t* = 0.29, df = 17, *p* > 0.05 by unpaired *t*-test), or time spent at the arm junction (Figure 6C; *n* = 8 Air and 11 EtOH, *t* = 0.97, df = 17, *p* > 0.05 by unpaired *t*-test), suggesting no difference in locomotion. Finally, we also assessed the number of times an animal engaged in a stretch attend posture (a measure of risk assessment) and found no significant differences between Air- and EtOH-exposed animals (Figure 6D; *n* = 8 Air and 11 EtOH, *t* = 0.43, df = 17, *p* > 0.05 by unpaired *t*-test).

## Discussion

**Figure 7:**
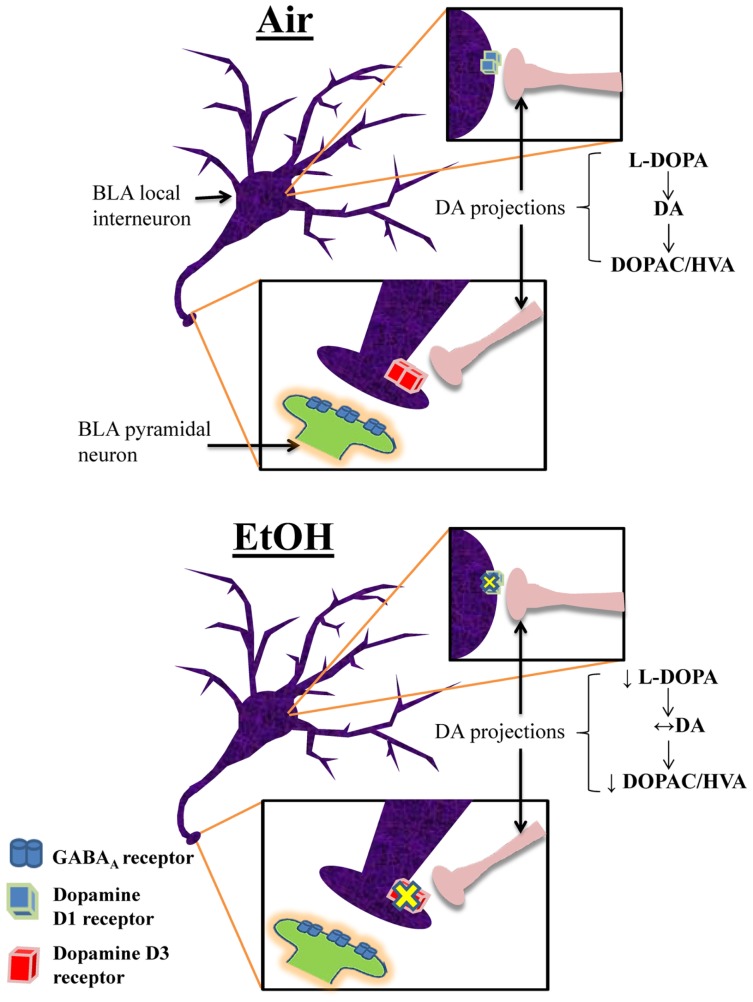
**Proposed model of the effects of third trimester-equivalent ethanol exposure on dopamine function in the BLA**. The top panel illustrates a local GABAergic interneuron inhibiting a pyramidal neuron in the BLA (postsynaptic pyramidal neuron dendrite only shown in zoomed in image). DA projections from the ventral tegmental area innervate local interneurons, releasing DA onto D1Rs and D3Rs (the exact location of DA innervation onto these local interneurons is unknown). In Air-exposed animals, D1R activation increases interneuron firing, resulting in increased action potential-dependent spontaneous GABA release. Conversely, D3R activation suppresses quantal action potential-independent release of GABA from interneurons. These two opposing effects regulate the balance of GABA transmission in pyramidal neurons. In EtOH-exposed animals, D1R and D3R function is significantly blunted, without changes in receptor levels. Furthermore, although l-DOPA levels are robustly reduced, DA levels remain unchanged due to decreased degradation of DA into DOPAC and HVA. These homeostatic changes presumably explain the normal behavior observed in the elevated plus maze.

This is the first characterization of the effects of moderate EtOH exposure during the third trimester-equivalent on the modulatory actions of DA in the BLA. We found that EtOH exposure significantly reduced the D1R-mediated enhancement of action potential-dependent spontaneous GABA_A_R-mediated transmission in pyramidal neurons. We demonstrated that EtOH exposure reduced D3R-mediated reduction of quantal GABA release at interneuron-to-pyramidal neuron synapses, which may represent a compensatory change aimed at restoring balance in the modulatory actions of DA in the BLA. We also found that EtOH exposure induced a decrease in the levels of the DA precursor, l-DOPA. However, DA levels were not significantly affected by EtOH and our findings suggest that this is a consequence of reduced degradation of DA. A model depicting these changes is shown in Figure 7. Importantly, anxiety-like behavior on the elevated plus maze was unaffected in EtOH-exposed rats, indicating that homeostasis was re-established in the BLA of treated animals.

### Moderate postnatal ethanol exposure reduces the D1R-mediated enhancement of sIPSC frequency and amplitude

In agreement with the literature (11, 26), D1R activation increased the frequency of sIPSCs in BLA pyramidal neurons from Air-exposed animals. This effect has been shown to be mediated by increased interneuron firing (11). We also found a DA-mediated increase in sIPSC amplitude that was blocked by the D1R antagonist that had not been previously reported. However, studies have either used a lower concentration of DA (11) or did not report sIPSC amplitude (26). It is possible that D1R activation could increase the duration of action potentials in interneurons, leading to an increase in sIPSC amplitudes in the pyramidal neurons (31, 32). Postsynaptic D1Rs (8, 11) may also directly potentiate GABA_A_Rs, although DA has been shown to not alter exogenous GABA-induced currents in mice (33). Another possibility is that at this concentration, DA may activate noradrenergic receptors, specifically α1 (28), which have been shown to increase GABA transmission in the BLA (29). Consistent with this, the α1 antagonist blocked the effect of DA on sIPSC amplitude, but not on sIPSC frequency. As previously mentioned, the D1R antagonist, SCH23390, also blocked the DA-induced increase in sIPSC amplitude. A likely explanation for this is that SCH23390 is a known blocker of inward rectifier K^+^ channels (34–36) and activation of α1-adrenoreceptors by DA can inhibit these channels (28). These data suggest that the effect of DA on sIPSC amplitude may be mediated by α1-adrenoreceptors. Future studies should examine interactions between the DA and noradrenergic systems in the BLA.

Importantly, the DA-mediated facilitation of GABA transmission was significantly blunted in peri-adolescent animals that were exposed to EtOH during the third trimester-equivalent. These alterations cannot be explained by changes in basal properties of sIPSCs as EtOH did not affect basal GABA transmission. Although western blot analysis indicated that total D1R levels within the BLA are not affected by EtOH exposure, a selective decrease in expression of D1Rs in local BLA interneurons could explain the change in D1R function and this should be further investigated. Other potential mechanisms that may result in decreased D1R function in the BLA include: (1) uncoupling of D1Rs from G proteins by a phosphorylation-dependent mechanism (37), (2) internalization of D1Rs (38), or (3) disrupted D1R-mediated signal transduction (37). It is worth noting that the small effect of TTX on sIPSC frequency (Figure 1) suggests that D1R activation engages a population of inputs that are normally silent under basal conditions. DA-positive terminals have been found to innervate parvalbumin- and calretinin-positive interneurons (10). Therefore, it is possible that under certain conditions where D1R activation is required to enhance GABAergic inhibition, DA release targets specific interneuron populations in the BLA. Future studies are necessary to determine the mechanism of action of EtOH on D1Rs, and potentially α1-adrenoreceptors, in the BLA.

### Moderate postnatal ethanol exposure abolishes the D3R-mediated decrease of mIPSC frequency

It has been shown that a selective D3R agonist inhibits quantal GABA release from local interneurons onto BLA pyramidal neurons (12), and we were able to replicate these findings using exogenous DA. Although the mechanism of this presynaptic effect is unclear, D3R activation can suppress extracellular signal-regulated kinase (ERK) activity (39), which can result in decreased GABA release (40). It is worth noting that a postsynaptic D3R effect (i.e., a reduction in mIPSC amplitude) was previously reported using the D3R agonist (12). It is possible that this postsynaptic D3R-mediated effect was masked by either a D1R-dependent or a potential α1-adrenoreceptor-mediated potentiation given that those receptors were not blocked in this set of experiments. However, given that the D3R effect on mIPSC amplitude was similar between the treatment groups, our findings suggest that postsynaptic receptors were not altered by the EtOH exposure.

Interestingly, the D3R-mediated suppression of GABA transmission was completely abolished in EtOH-exposed animals. Together with a deficit in D1R-mediated enhancement of GABA transmission, these findings suggest that homeostatic changes occurred in the DA system. Specifically, EtOH exposure may have decreased function of one of these DA receptors, and the system decreased function in another DA receptor that exerts an opposite effect on GABA release, perhaps as a compensatory mechanism to maintain stable GABAergic inhibition. Based on our findings, it is difficult to conclude which was first impaired by EtOH exposure. However, a human study reported that infants exposed to EtOH through the third trimester showed reduced alert and attentive states during testing (41), which may be an indicator of suppressed amygdala activation. Fetal EtOH-exposed infants exhibited slower registration of auditory and visual stimuli (42), indicating deficits in sensory processing, which may contribute to impaired behavioral arousal. Moreover, a recent study demonstrated that fetal EtOH exposure was robustly associated with higher infant emotional withdrawal (43), which can lead to altered amygdala development (44). Deficits in D3R function in the BLA would result in over-inhibition of this brain region and could explain these behavioral alterations, suggesting that D3Rs could be a primary target of EtOH early in development. Additionally, studies are required to further understand the effects of third trimester-equivalent EtOH exposure on the development of the DA system in the BLA.

Western blot analysis also showed that total D3R expression in the BLA was unaltered by EtOH exposure. However, mechanisms similar to those described for alterations in D1R function may explain the loss of D3R function: (1) uncoupling of D3Rs from G proteins by a phosphorylation-dependent mechanism (45–47), (2) internalization of D3Rs (38), or (3) interrupted D3R-mediated signal transduction (48). These possible explanations require further investigation.

### Moderate postnatal ethanol exposure reduces the levels of l-DOPA, DOPAC, and HVA

DA synthesis is initiated by conversion of tyrosine to l-DOPA by tyrosine hydroxylase, which is then converted to DA by DOPA decarboxylase (Figure 5A). After release, DA is taken up into the terminal where it can be degraded by monoamine oxidase (MAO) to produce DOPAC, which can be further catalyzed by catechol-*O*-methyltransferase (COMT) into HVA [Figure 5A; also reviewed by (49)]. Interestingly, we found that l-DOPA levels were significantly decreased in EtOH-exposed animals, suggesting that EtOH exposure decreases DA synthesis. This is consistent with a study showing that young adult rhesus monkeys exposed to EtOH in mid-to-late gestation exhibited suppressed DA synthesis (50). Reduced DA synthesis could occur to compensate for repeated EtOH-induced increases in DA release during EtOH exposure (51).

Despite the decreased levels of l-DOPA, DA levels were surprisingly unaltered in the BLA of EtOH-exposed offspring. However, levels of DOPAC and HVA were significantly reduced in EtOH-exposed animals, suggesting that MAO and COMT activity is also reduced, perhaps to compensate for reduced DA synthesis. Studies have reported similar decreases in levels of HVA in the cerebrospinal fluid of rhesus monkeys prenatally exposed to EtOH (52) and in the striatum and frontal cortex of rats exposed to EtOH *in utero* (53). Furthermore, a recent study found that exposure to a high dose of EtOH during the second trimester-equivalent reduced MAO activity and HVA levels in fetal whole brain, with no changes in DA levels (54). It is also possible that this represents a compensatory mechanism in response to reduced D1R and D3R function, or that reduced DA receptor function could follow changes in DA transmission. Importantly, these findings demonstrate another homeostatic change in the BLA aimed at restoring homeostasis in the DA system following EtOH exposure during the third trimester-equivalent. One caveat about these findings is that these analyte levels were crude measures of tissue content. It would be interesting to further characterize these neurochemical changes using microdialysis and fast scan cyclic voltammetry.

### Moderate postnatal ethanol exposure did not affect anxiety-like behavior

EtOH-exposed animals did not exhibit alterations in anxiety-like behavior compared to Air-exposed animals on the EPM. Similar findings were shown on an open field in rats exposed to EtOH from P1-P7 via intra-gastric gavage (55). Conversely, exposure to EtOH during the first and second trimesters has been shown to increase anxiety-like behavior (56–59), particularly in response to stress (60). This is generally consistent with human studies as exposure time-dependent effects on anxiety-like behaviors have been suggested to occur in children up to 8 years old that were prenatally exposed to EtOH. Specifically, moderate-to-high EtOH exposure in the first trimester leads to increased odds of anxiety while heavy exposure to EtOH late in pregnancy reduces the odds of anxiety in children prenatally exposed to EtOH (16). Although the molecular or physiological alterations underlying these differences are not clear, exposure during early gestation appears to decrease basal GABA transmission in the BLA (57) and increases dendritic spines on apical dendrites of pyramidal neurons within the BLA (59). In contrast, our study indicates that basal GABA transmission in the BLA is not altered following exposure during the third trimester-equivalent.

DA levels rise in the BLA in response to stressful and/or novel stimuli (5, 6), and DA antagonists alter anxiety-like behaviors. Physiologically, D1R activation enhances inhibition in pyramidal neurons [current study and (11, 26)] presumably to suppress over-excitability of the BLA and behavioral arousal. Consistent with this, a number of behavioral studies have demonstrated that microinjection of D1R antagonists into the BLA results in anxiogenic responses (13). In contrast, D3R activation can disinhibit pyramidal neurons [current findings and (12)], thereby increasing BLA excitability and behavioral arousal. This is supported by behavioral studies that have demonstrated that microinjection of D3R antagonists into the BLA reduce anxiety-like behaviors (12) and taste-related associative learning (61). Furthermore, D3R knockout mice exhibit anxiolytic behavior (14). Taken together, it is clear that D1Rs and D3Rs in the BLA play opposing roles in modulation of anxiety-like behaviors. However, given the compensatory mechanisms in both DA receptor function and DA neurochemistry following EtOH exposure, it is not surprising that EtOH-exposed animals did not exhibit alterations in anxiety-like behavior. It is possible that exposure to higher levels of EtOH could overcome these compensatory mechanisms. Future studies are necessary to assess this possibility.

## Conclusion

It has been hypothesized that fetal exposure to EtOH leads to hypofunction of the DA system (30, 62–65). DA hypofunction has been shown across species and with exposure occurring at different gestational periods (30), suggesting that this neurotransmitter system is particularly sensitive to EtOH in some brain regions. However, our study suggests that compensatory mechanisms are engaged in the DA system within the BLA to restore anxiety-like responses following exposure to moderate EtOH during the equivalent of the human third trimester, a period when pregnant women often consume EtOH (15). Interestingly, there are other reports of homeostatic changes following developmental EtOH exposure in the cerebellum (66), hippocampus (67–69), and medial septum (70). Future studies should investigate whether under certain conditions (e.g., binge-like heavy EtOH exposure) compensation may fail, contributing to behavioral deficits (71).

## Conflict of Interest Statement

The authors declare that the research was conducted in the absence of any commercial or financial relationships that could be construed as a potential conflict of interest.
